# A New Type of Liquid Silymarin Proliposome Containing Bile Salts: Its Preparation and Improved Hepatoprotective Effects

**DOI:** 10.1371/journal.pone.0143625

**Published:** 2015-12-16

**Authors:** Mei Wang, Tingting Xie, Zhanying Chang, Ling Wang, Xiangyun Xie, Yaohong Kou, Hongxia Xu, Xiaoli Gao

**Affiliations:** 1 Department of pharmaceutics, College of Pharmacy, Xinjiang Medical University, Urumqi, China; 2 Department of Pharmaceutical Care, PLA General Hospital, Beijing, China; Aristotle University of Thessaloniki, GREECE

## Abstract

Silymarin, a known extract, is used in the treatment of liver diseases with various origins, but its current administration form cannot target the liver because of its poor oral bioavailability. A new type of oral silymarin proliposome aimed at improving silymarin’s poor bioavailability and hepatoprotective effects, is introduced in this work. Silymarin-loaded liquid proliposome were prepared using a simple dissolving process. The morphology, particle size, zeta potential, and entrapment efficiency of the silymarin liposomes were analysed. The everted gut sac transport model was used to measure the intestinal transport of liposomes. The liposomal hepatoprotective activity was evaluated in three types of experimental hepatitis animal models. After staining with haematoxylin and eosin, the livers were microscopically examined to analyse any pathological changes. The prepared silymarin proliposome formed silymarin liposomes with a multilayer liposome structure and improved intestinal transport. In an injured liver, the silymarin liposomes produced a stronger hepatoprotective effect through a significant decrease in both the aminotransferase and MDA levels and a significant increase in the SOD and GSH-PX levels compared to orally administered silymarin tablets. This effect was also confirmed histopathologically. In a word, incorporation of silymarin into a liposomal carrier system increased intestinal absorption and showed better hepatoprotective effects compared to silymarin tablets.

## Introduction

Silymarin is the main active flavonoid extract in the dried fruits of S. marianum and has been widely used in the treatment of various liver diseases. Silymarin is a complex mixture of the following four flavonolignans: (1) silybin, (2) isosilybin, (3) silydianin, and (4) silychristin. Among these compounds, silybin is the major and most active component of silymarin; it is responsible for silymarin’s pharmacological activity. Silymarin has many advantages for treating liver disorders, including its ability to inhibit hepatotoxin binding to receptor sites and to protect the liver against injury, its ability to reduce glutathione oxidation to enhance its own levels in the liver, and its ability to stabilize the cell membrane of the liver and increase hepatocyte protein synthesis. Furthermore, silymarin can be used to prevent diabetes syndrome and atheroma by decreasing blood lipids and inhibiting platelet aggregation, etc[1]. The drug is well tolerated and has relatively few adverse effects. However, the effectiveness of silymarin has been discounted due to its poor water solubility and low bioavailability after oral administration[2]. The absorption of silymarin by the gastrointestinal tract is only between 20–50% due to its poor water solubility and partial degradation by gastric fluid, both of which limit its application[3]. To overcome these problems and improve silymarin’s bioavailability, many approaches have been investigated, including incorporating it into solid dispersion systems[4];forming polyhydroxyphenyl chromanone salts, soluble derivatives and complexes with phospholipids[5]; encapsulating it into liposomes[6,7]; and solubilising it in self-microemulsifying drug-delivery systems[8,9]and nanoparticles[10,11].

Liposomes have been able to improve the oral absorption and bioavailability of hydrophobic drugs. Liposomes consisting of lipid bilayers with an internal water phase can encapsulate both water-soluble and lipophilic drugs. Liposomes can improve drug stability and bioavailability, can target specific sites, and can control in vivo behaviour by modifying the drug-surface properties; thus, liposomes have been widely studied for use in targeted drug-delivery systems, and several liposomal products have been marketed[12,13,14]. However, liposomes continue to lack a significant medical impact despite a relatively long history of investigation. Limited physical and biological stability, including aggregation, sedimentation, fusion, phospholipid hydrolysis, and/or oxidation, have limited its development. For many years, there have been attempts to develop liposomes, including making proliposome, which is a dry, free-flowing powder that can form liposomes upon reconstitution. Because these formulations exist as a dry powder, proliposomes are both far more stable and more suitable for oral delivery than liposomes. Therefore, proliposomes are a potential vehicle to help improve the oral absorption of hydrophobic drugs[15]. However, it is difficult to scale up the production of proliposomes because of the complex techniques required to make them[16].

Liquid proliposomes have been reported in several articles. They have been reported as a type of transparent solution that forms drug-loaded liposomes when blank proliposomes are mixed with water or a 0.9% NaCl aqueous solution containing the drug[17,18,19]. Compared to that of solid proliposomes, the preparation process of liquid proliposomes is simpler and requires no specialised devices. Additionally, drug-containing liposomes can automatically form within a short time without applied force, such as sonication or extruders, when the liquid proliposomes make contact with a water phase. The reported proliposomes are often made by first dissolving a mixture of phospholipids and the drug in dehydrated alcohol, forming a light-yellow, transparent liquid referred to as the proliposomes. Then, the hydrated liposomes are formed automatically by dropping distilled water into the proliposomes and shaking the mixture manually for several minutes. These proliposomes can be stored until use without any liposomal physical and biological stability problems because there is no water in their formula. Their preparation is so simple that complicated devices are not required, and thus, their production cost is low. Additionally, as reported, sodium deoxycholate incorporated into the proliposomal formulation can facilitate liposome formation so that lipids containing bile salts can readily transform into vesicular or mixed micelles in the gastrointestinal environment[20, 21, 22]. However, the use of ethanol as a solvent in this proliposomal method has some disadvantages because of its high volatility and pharmacological effects.

Based on these findings, the proliposome method was modified and developed in our laboratory. Unlike the reported proliposomes, our proliposomes do not use ethanol as a solvent so that we avoid its disadvantages; instead, they use a nonaqueous solvent such as propylene glycol, PEG-400, ethyl lactate, etc., according to the drug’s solubility. Furthermore, the effect of sodium deoxycholate in the formulation was investigated. Sodium deoxycholate and sodium oleate were compared in the formulation. We observed no differences between sodium deoxycholate and sodium oleate when producing the proliposomes. Thus, our proliposomes are composed of the drug, soybean lecithin, and cholesterol, as well as sodium oleate, sodium deoxycholate, or poloxamer-88. The yellow proliposome liquid solution can be made by dissolving all of the solid components into a nonaqueous solvent. In previous work in our laboratory, the anticancer drug tegafur was successfully encapsulated into liposomes with well-distributed particle sizes, good absorption, high bioavailability, and selective tissue distribution and relative bioavailability compared to a drug solution at 251.8%[23].

In this study, the highly hydrophobic compound silymarin was trapped into liposomes to improve its solubility and absorption. The morphology, mean particle size, zeta potential, and entrapment efficiency of the silymarin proliposomes were investigated after the silymarin liposomes were formed. The absorption in vitro and hepatoprotective activity against acute hepatitis induced three ways in mice were also investigated.

## Materials and Methods

### Materials

Silymarin was obtained from the Jian Herbal Extract Factory (Batch No. 000419, Jilin, China) with a total flavonolignan purity of >74%, as determined by high-performance liquid chromatography (HPLC). The silybin standard was purchased from the National Institute for the Control of Pharmaceutical and Biological Products (Beijing, China). Soy lecithin was purchased from the Jinban Pharmaceutical Company (Shanghai, China). SephadexG-50 was provided by the Chinese Medical Reagent Company (Shanghai, China). Silymarin tablets were obtained from the Jiuhui Pharmaceutical Company (Specs: 77 mg/tablet Jiuhui City, China). D-Galactosamine was purchased from the Biomedical Institute of Chongqing Medical University (Batch No. 050326, Chongqing, China). Superoxide dismutase (SOD), malondialdehyde (MDA), and glutathione peroxidase (GSH-PX) were obtained from the Jiancheng Bioengineer Institute (Nanjiang, China). Ten Wistar rats with similar body weights (250–300 g) and 240 kunming mice (18–20 g) were selected from the Xinjiang Medical University in accordance with the animal experiment regulation. All of the other chemicals were reagent grade.

### Preparation of silymarin proliposomes

Silymarin proliposomes were prepared using a new proliposome method, as previously described by the authors[24]. Briefly, 1.0 g silymarin, 10.0 g soy lecithin, 5 g cholesterol, and 0.5 g sodium oleate were dissolved in 100.0 mL propylene glycol until a light-yellow transparent solution was formed, which is referred to as the silymarin proliposome solution. Then, 1 mL proliposome solution was mixed with distilled water. A liposomal suspension was obtained by shaking the mixture manually because the soy lecithin would automatically array the solution into a lipid multilayer structure and subsequently entrap the silymarin into liposomes[24].

### Particle size, zeta potential, and morphology of silymarin proliposomes

The samples were hydrated with distilled water for size measurements. After adding various amounts of distilled water into 1 mL of the proliposomes, silymarin liposomes at various diluted concentrations—1:50, 1:100, and 1:250—were formed separately by gentle shaking. Then, the mean particle size and particle size distribution of silymarin-loaded liposomes were measured using a dynamic scattering instrument (Nano-2490 particle sizer, Malvern, UK). The proliposomes were similarly hydrated in distilled water to meet the zeta potential determination requirements of the instrument. The results are expressed as the mean ± standard deviation for at least three different batches of each liposome formulation. The morphology of the liposomes was observed using a transmission electron microscope (TEM) (H-600, Japan) after being diluted to 1:50. One millilitre of liposomes was diluted with 50 mL distilled water and dropped onto a carbon-coated copper grid, forming a thin liquid film. The sample excess was then removed using filter paper. The films on the grid were allowed to dry for several hours, and then they were observed using the TEM and imaged.

### Drug entrapment efficiency of silymarin-loaded liposomes

Various amounts of lecithin and a constant amount of drug were used in the formulation to prepare silymarin proliposomes with various drug-to-lecithin mass ratios. Next, 1 mL silymarin proliposome solution was drawn and mixed with 20 mL distilled water to form silymarin-loaded liposomes. The percentage of silymarin encapsulated within the liposomes was determined using a gel-filtration chromatography method, as described above[24, 25]. Briefly, 1 mL of hydrated silymarin liposomes were eluted from Sephadex, a G-50 column (pre-saturated with empty liposomes), using phosphate buffered saline (PBS) of pH 7.2 with a flow rate of 1 mL/min[26]. Next, the opalescent part of the eluate was collected. The silymarin in the eluate and in the suspensions was analysed with a UV-spectrophotometer (UV- 2401, Shimadzu, Japan) at 288 nm, which is the wavelength of the maximum absorption of silymarin in methanol. The concentration of silymarin can be mathematically calculated with a standard curve. The encapsulation efficiency (EE%) of silymarin in the liposomes was calculated using the following formula:

$$EE(\%)=\frac{\mathrm{Entapped drug amount}(\mathrm{mg})}{\mathrm{Total amount added}(\mathrm{mg})}\times 100\%$$

Silymarin proliposomes with drug-to-lecithin mass ratios of 1:5, 1:10, and 1:20 were prepared separately, and the silymarin entrapment efficiency was determined according to the above method to investigate the effect of the drug-to-lecithin mass ratio on the encapsulation efficiency. Then, 1 mL silymarin proliposome with a 1:10 drug-to-lecithin mass ratio was hydrated with 20 mL phosphate buffered solution of pH 5.0 and adjusted to pH 5.0, 6.0, 7.0, and 9.0 with a 1 M sodium hydroxide solution (NaOH) to study the influence of pH on EE%. The EE% values of silymarin liposomes with different drug-to-lecithin mass ratios and different pHs on the first day and the third day after preparation were also compared to investigate the liposome stability.

### Ethics statement

All of the animal studies were conducted in strict accordance with the recommendations in the Guide for the Care and Use of Laboratory Animals. The protocol was approved by the Committee on the Ethics of Animal Experiments of Xinjiang Medical University (animal certification Number: Shengchanxuke(xin)2011-0003). All surgery was performed under sodium pentobarbital aesthesia, and all efforts were made to minimize suffering.

### Everted rat intestinal sac transport studies

One millilitre of silymarin proliposomes was added to 10 mL of distilled water to form the silymarin liposomes that were used for the silymarin liposome group. The same amount of silymarin that was used to prepare the silymarin proliposomes was dissolved in propylene glycol to form a solution, and then 1 mL of the solution was added to 10 mL of distilled water to produce the silymarin control solution. The everted gut sac transport experiments were performed in triplicate at 37°C in a water bath. On the day of the experiment, Wistar rats were anesthetised and euthanized using sodium pentobarbital injection, a midline abdominal incision was made, and the entire length of the intestine was removed; then, the first 15-cm segment distal to the pylorus was taken. The excised intestine was flushed with ice-cold saline to remove any intestinal content and the intestinal segment was everted on a thin glass rod. The everted intestine was ligated on one end and infused with the serosal fluid, and then the other end was also ligated. The full length of the everted intestine was 10 cm. Next, the everted intestine was placed into 25 mL of silymarin solution or silymarin liposomes, which served as the mucosal fluid (outer compartment). Six sacs were made. One everted intestine was removed, and 0.5 mL samples of serosal fluid were withdrawn at fixed time points for 1 hour. The diffusion apparatus was kept in a constant-temperature shaking bath at 37°C with a continuous supply of 95% oxygen throughout the experiment. The drawn samples were analysed using UV. The experiment was performed in triplicate, and the mean of three samples was used in the data analysis. The area under the absorption curve (AUC) was calculated via the trapezoidal method and compared with the silymarin solution[27, 28].

### Hepatoprotective activity test of silymarin liposomes on acute experimental hepatitis

To investigate the hepatoprotective activity of silymarin liposomes on differently induced acute experimental hepatitis, the following three experimental hepatitis animal models were established: (1) a model induced by intraperitoneal injection of carbon tetrachloride (CCl_4_) in mice, (2) a model induced by intraperitoneal injection of D-galactosamine (D-GalN), and (3) a model established by a caudal vein injection of Bacillus Calmette-Guerin and lipopolysaccharides (BCG+LPS) in mice. In each model, the animals were divided into eight groups of ten mice. Group 1 was kept as a control group. The mice in the remaining seven groups received carbon tetrachloride (0.1%,10 mL/kg) intraperitoneally (i.p.) for the first model, D-galactosamine D-GalN (1000 mg/kg) i.p. for the second model, and Bacillus Calmette-Guerin and lipopolysaccharides (BCG+LPS) (0.1 mL in each mouse) by caudal vein injection for the third model to induce hepatic damage. To minimize tissue damage, the smallest possible needle was used. Group 2 did not receive silymarin and acted as an untreated group. Groups 3, 4, and 5 received silymarin tablets by intragastric administration (75, 150, or 300 mg/kg·d separately) twice daily every day. Groups 6, 7, and 8 received silymarin liposomes via the same method (75, 150, and 300 mg/kg·d separately) every day. For the CCl_4_-induced model and the D-GalN-induced model, the silymarin tablet and liposome administration was started six days prior to CCl_4_ or D-GalN injection and continued until the end of the experiment. For the BCG+LPS-treated model, the BCG solution was also administered from the first day to the eleventh day along with silymarin tablets and liposomes; on the twelfth day, LPS saline solution (7.5 μg/0.2 mL) was administered by caudal vein injection to each mouse immediately after the silymarin liposome and tablets were given by gavage. During the drug treatment, the animals were housed in a 12-hr light/dark cycle at a controlled room temperature and had free access to food and water. The experiments were performed during the light cycle. Each mouse in each group was weighed every other day, and its activity was observed to ensure that each mouse was in good condition. Furthermore, no unintended deaths of any animals occurred during this study.

Twenty-four hours after the three models were induced, all of the animals were sacrificed using cervical vertebra dislocation. Just before the sacrifice, blood samples were procured under sodium pentobarbital anaesthesia via retro-orbital bleeding. For each mouse, the blood was collected, and the serum was separated. The collected serum was biochemically tested for transaminase levels for both alanine aminotransferase (ALT) and aspartate aminotransferase (AST). The liver of each mouse was immediately removed. The left part of the liver was fixed in 10% formalin, serially sectioned, and microscopically examined after staining with haematoxylin and eosin to analyse any pathological changes. The right section of the liver was made into a homogenate and was used to measure malondialdehyde (MDA) levels, superoxide dismutase (SOD) levels, and glutathione peroxidase (GSH-PX) activities; the nitric oxide (NO) content was measured only in the BCG+LPS-induced group. Liver pathological injury was observed. The effects of the silymarin liposomes on all of the biochemical parameters above were observed and compared with silymarin tablets[29].

### Statistical analysis

All of the data were expressed as the mean ± SD. Statistical significance was determined by Student’s t-test except for the hepatoprotective activity test results, which were analysed by the q-test. Data were considered to be significant at P<0.05, unless otherwise indicated.

## Results and Discussion

### Particle morphology, particle size, and zeta potential

In this study, we observed that the mean particle size of liposomes hydrated from liquid proliposomes was small (approximately 70 nm) with a homogenous, narrow size distribution (Table 1), which is smaller than the reported particle size (378.6±26.5 nm) [30]. This indicates that liposomes can be formed easily with our method. Table 1 also shows that the particle size differed when 1 mL of silymarin proliposome was mixed with various amounts of water. The reason for the difference might be that the hydration degree was different when different amounts of water were added. All of the particle sizes were below 100 nm when the proliposomes were diluted with various amounts of water. The zeta potential was approximately ‒31.54 mv when the proliposomes were diluted to 1:50.

**Table 1:** Mean particle sizes and zeta potentials of silymarin liposomes at different dilutions with water ratios (n = 3).

	Mean particle sizes (nm)			Ξ potential (mv)
Diluted times	1∶50	1∶100	1∶250	
Silymarin liposome	55.92±8.42	46.43±10.65	75.83±11.62–31.54±10.13	

**Fig 1:**
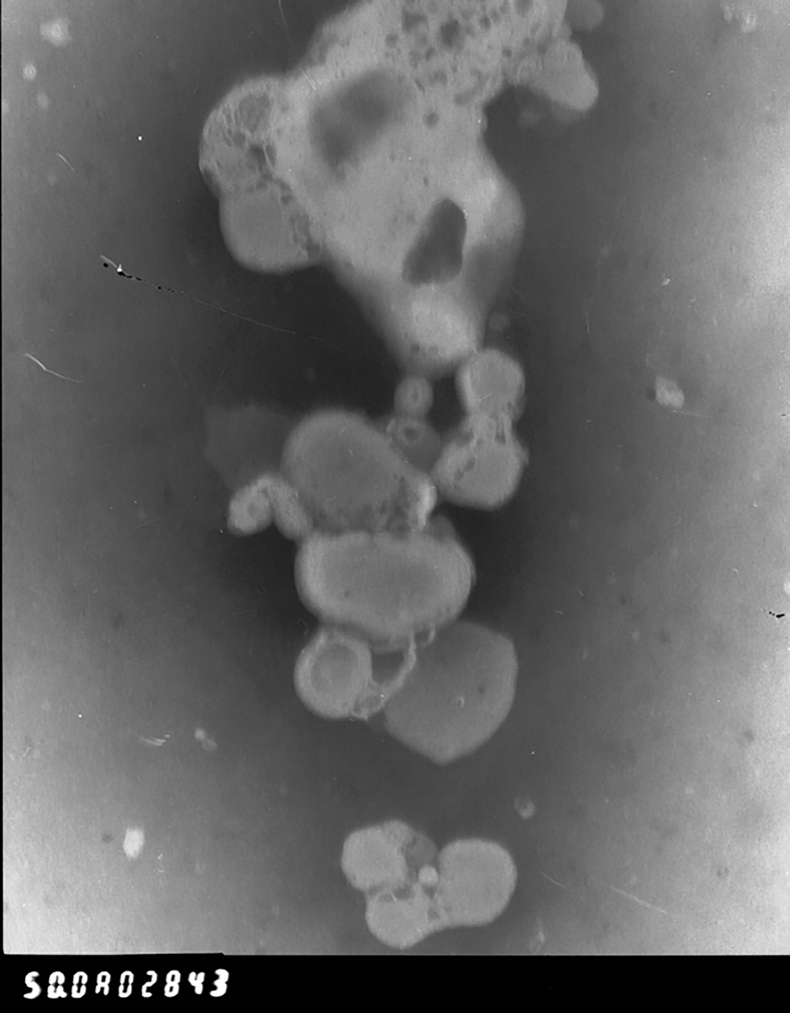
Transmission electron images of silymarin loaded liposomes (5×1000 times).

We also observed the morphology of the silymarin-loaded liposomes as imaged by transmission electron microscopy, which is shown in Fig 1. The silymarin liposomes formed were round with multiple layers, which confirms the formation of liposomes from proliposomes. Additionally, the incorporation of sodium deoxycholate into the formulation could facilitate liposome formation. In this study, we compared the morphology of liposomal formulation with or without sodium oleate and found no obvious differences in their morphologies.

### Entrapment efficiency of liposomes

The drug encapsulation efficiencies and drug loading efficiencies with various mass ratios of silymarin to phospholipid are shown in Table 2. The results reveal that the mass ratio of drug to phospholipid (D/PC) has a clear effect on the drug-entrapment efficiency and drug loading efficiencies. There is a significant increase in EE% from 30.66±9.62% to 42.17±2.24% when the drug-to-phospholipid ratio was increased from 1:5 to 1:10 (*P*<0.01); however, the entrapment efficiency was mostly unchanged after increasing the ratio to 1:20 (*P*>0.05) while drug loading efficiency decreased. The increased entrapment efficiency along with an increased amount of phospholipid with a certain limit might indicate that increasing appropriate amounts of phospholipid could be helpful for increasing drug encapsulation into the liposomes. Too large amount of phospholipid did not improve the drug-encapsulation efficiency but decrease the drug loading efficiency. Thus, the optimal drug-to-phospholipid mass ratio was determined to be 1:10.

**Table 2:** Silymarin entrapment efficiency with various drug-to-lipid ratios (EE%) (n = 3).

	Drug / Phospholipid *(W/W)*		
	1∶5	1∶10	1∶20
EE%	30.66±9.62	42.17±2.24*	44.14±5.53**
% drug loading	10.14±3.18	7.10±0.38	4.25±0.53

^*^ *P*<0.01 *vs* Drug/Phospholipid = 1:5 group

^**^ *P*>0.05 *vs* Drug/Phospholipid = 1:10 group.

The entrapment efficiency also changed with changes in pH, as shown in Table 3. When the pH of the liposome suspension with a drug-to-phospholipid ratio of 1:10 was adjusted to pH 5.0, 6.0, 7.0, and 9.0, respectively, the drug-entrapment efficiency increased correspondingly with increasing pH, which demonstrated that a larger quantity of the drug was easily entrapped when the liposome suspension was in a more alkaline environment. The reason may be connected to the structure of silymarin and its interaction with the phospholipid.

**Table 3:** Silymarin entrapment efficiency at various pH values after three days of storage (n = 3).

Time (day)	Drug/phospholipids = 1:10				Drug/phospholipids	
	pH 5.0	pH 6.0	pH 7.0	pH 9.0	1:05	1:20
First day	28.39±3.65	35.11±4.39	42.17±2.24	52.69±1.69	36.00±3.76	45.84±6.62
Third day	28.99±4.95	35.21±6.20	42.43±3.10	53.38±1.85	30.66±9.62	44.14±5.53

Furthermore, an encapsulation efficiency of 40–50% is an acceptable level for the silymarin-loaded liposomes in this study, especially when compared with other studies in which silymarin’s encapsulation efficiencies for much larger liposomes (i.e., 145–168 nm) have only been approximately 60%[7].

Most liposomes can aggregate or fuse upon storage, resulting in decreased drug-loading efficiency. However, silymarin proliposomes can be easily stored at room temperature or 4°C without any change in particle size or drug entrapment efficiency. Furthermore, even when silymarin-loaded liposomes are formed after hydration with distilled water, they show good stability at room temperature. Table 3 also shows that the entrapment efficiency was unchanged after liposomes were stored for three days at room temperature, regardless of drug-to-PC ratio or pH changes. This finding shows that silymarin liposomes can be stable for three days at room temperature and that the drug leakage ratio will be low.

Although the entrapment efficiency is not significantly high, our study proves that silymarin can be incorporated into liposomes, as expected, and that its effects can be seen in the following transport and hepatoprotective-activity studies.

### Everted rat sac transport studies

Everted rat sac transport experiments provide an estimate of drug absorption via an in vitro method. This method is a robust and reproducible assay that is relatively fast (<2 h), inexpensive and straightforward. The in vivo area under the curve could be estimated using the permeation curve, which provides indicators of drug absorption and bioavailability in vivo. In this study, the formula containing 1 g drug, 10 g phospholipid, 5 g cholesterol, and 0.5 g sodium oleate was chosen because it had the best liposome-entrapment efficiency. The results are shown in Tables 4 and 5, which indicate that the silymarin liposome group had better transport, with an approximately 1-fold increase in AUC compared to the control group (*P*<0.01).

**Table 4:** Concentration changes in the serosal fluid (n = 5).

Time (min)	Conc. of serosal fluid (μg/ml)	
	Silymarin liposome	Silymarin control solution
10	0	0
20	21.21±0.77	6.32±0.10
30	34.07±1.83	17.15±0.78
40	54.67±2.73	34.96±1.64
50	95.97±3.18	47.93±1.11
60	124.05±1.25	91.74±1.79

**Table 5:** Area under the curve (AUC) of serosal absorption (n = 5).

Sample	AUC (μg/ml)*min (×10^3^)
Silymarin liposome	2.679
Silymarin control solution	1.522

It has been shown that liposomes can be absorbed through the lymphatic and M cells in the intestine and that soy lecithin can improve drug absorption. Thus, many drugs have been made into liposomes to improve their bioavailability[31]. Because of its poor water solubility, silymarin does not possess high bioavailability. In the everted rat sac transport studies, the increased transport might be due to the transport of the proliposomal drug through the M cells and the effect of the lecithin. Therefore, the results prove that this proliposomal formula might serve as a better carrier than other oral dosage forms for silymarin because they have shown a greater ability to transport drugs through the intestine.

### The hepatoprotective effects of silymarin liposomes on experimentally induced hepatitis

**Fig 2:**
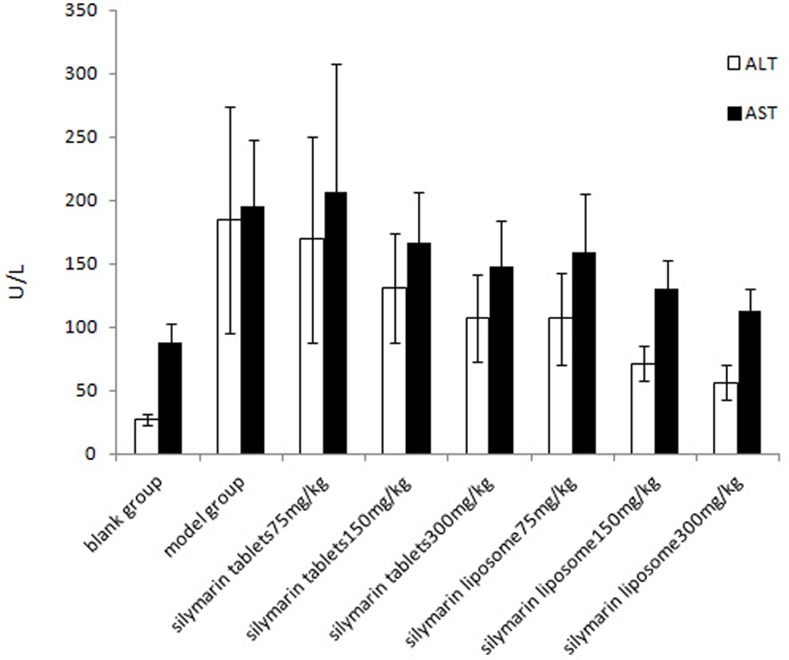
Effect of silymarin liposome on ALT, AST in experimental hepatitis animal model induced by CCL4.

**Fig 3:**
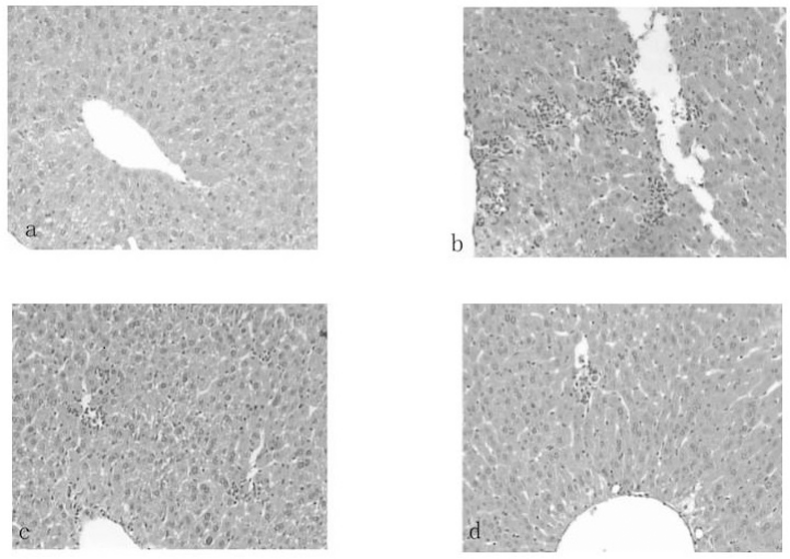
Photomicrograph (100×) of histological sections(hematoxylin and eosion stained)representing liver of mice treated with (a) saline; (b) CCl4; (c) CCl4 and received oral silymarin tablets with 300 mg/kg·d, (d) CCl4 and received oral silymarin liposomes with 300 mg/kg·d.

**Fig 4:**
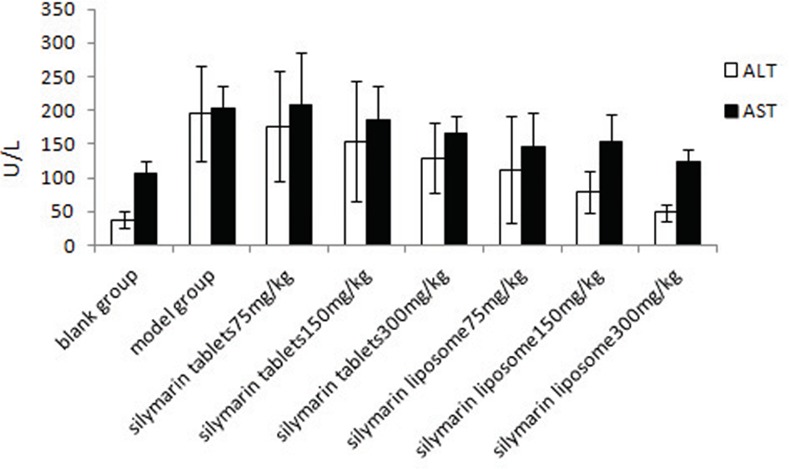
Effect of silymarin liposome on ALT, AST in experimental hepatitis animal model induced by D-GalN.

**Fig 5:**
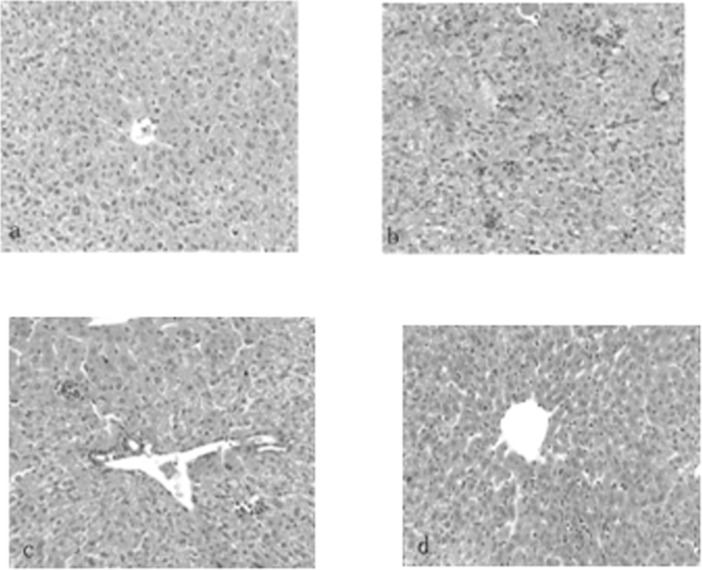
Photomicrograph (100×) of histological sections(hematoxylin and eosion stained)representing liver of mice treated with (a) saline; (b) D-GalN; (c) D-GalN and received oral silymarin tablets with 300 mg/kg·d, (d) D-GalN and received oral silymarin liposomes with 300 mg/kg·d.

**Fig 6:**
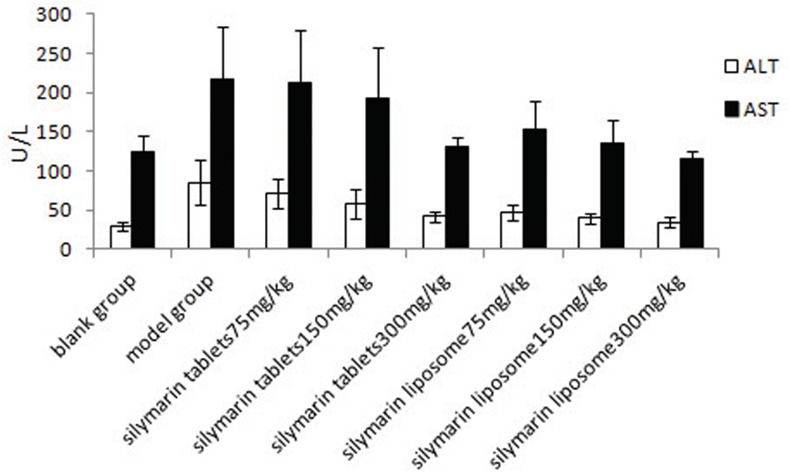
Effect of silymarin liposome on ALT, AST in immunological hepatitis animal model induced by BCG+LPS.

**Fig 7:**
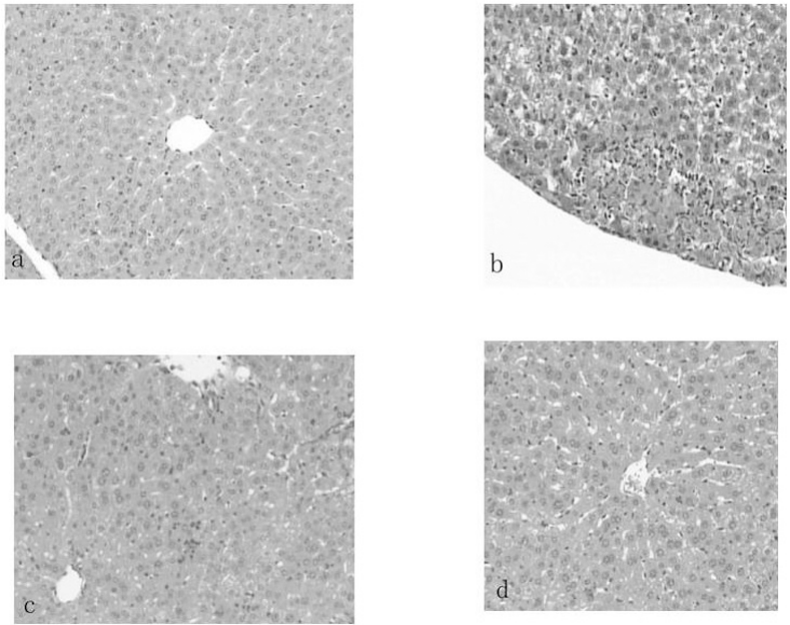
Photomicrograph (100×) of histological sections(hematoxylin and eosion stained)representing liver of mice treated with (a) saline; (b) BCG+LPS; (c) BCG+LPS and received oral silymarin tablets with 300 mg/kg·d, (d) BCG+LPS and received oral silymarin liposomes with 300 mg/kg·d.

In the experimental hepatitis animal model induced by CCl_4_, acute CCl_4_ administration resulted in a significant (*P*<0.01) decrease in GSH-PX to 78.74±23.20 U/mg prot and SOD to 53.56±10.04 U/mg prot and an increase in MDA to 1.99±0.61 nmol/mg prot compared to the normal values, which were 184.78±33.24 U/mg prot, 81.02±10.14 U/mg prot, and 1.29±0.34 nmol/mg prot, respectively (Table 6). Similarly, CCl_4_ administration also increased the serum ALT and AST levels sharply (*P*<0.01) compared to the normal values (Fig 2). Administration of silymarin liposomes significantly decreased the ALT and AST levels in serum in a dose-response manner compared to the CCl_4_-administered group (*P*<0.01) (Fig 2). The liposomes also prevented the reduction in the GSH-PX and SOD levels and decreased the MDA content in the liver homogenate compared to those for the CCl_4_-administered group (*P*<0.01) (Table 6). The effects of silymarin liposomes on the ALT and AST levels in serum and the SOD and GSH-PX activities in the liver homogenate are more effective than those of silymarin tablets at the same doses *(P<*0.05) (Fig 2). Pathological observations of the liver show that the liver injury of silymarin liposomes administered at 300 mg/kg·d is significantly less than that of the model and silymarin-tablet groups. The massive area of necrosis, hepatic lesions, inflammatory cells, ballooning, and vacuolar degeneration of hepatocytes was remarkably reduced by the oral administration of silymarin liposomes (Fig 3). Similar results were observed in the experimental hepatitis animal model induced by D-GalN. Silymarin liposomes significantly reduced the ALT and AST levels in serum (*P*<0.01) (Fig 4), increased the SOD and GSH-PX activities, and decreased the MDA content in the liver homogenate (*P*<0.01), especially when larger doses were administered (Table 7). The effects of silymarin liposomes on the ALT levels in the serum and the GSH-PX levels in the liver homogenate are more obvious compared to those for the silymarin tablets, whereas there were not large changes in the ALT, SOD, and MDA levels between the two groups (Fig 4 and Table 7). The liver histopathological observations showed that the silymarin liposome groups had more positive effects on liver injury compared to the silymarin tablets when larger doses (300 mg/kg·d) were administered (Fig 5). In the animal model induced by BCG+LPS, silymarin liposomes significantly decreased the ALT and AST levels in the serum (Fig 6), reduced the NO content, increased the SOD and GSH-PX activities, and decreased the MDA content in the liver homogenate compared to those for the BCG+LPS-treated group (*P*<0.01) (Table 8). Additionally, the administration of silymarin liposomes produced stronger biological effects than the administration of the silymarin tablets for all of the administered doses (*P*<0.05) (Table 8). The liver pathological observations showed that the massive area of necrosis and hepatic lesions induced by BCG+LPS were remarkably more reduced by the administration of oral silymarin liposomes (300 mg/kg·d) than by silymarin tablets (Fig 7).

**Table 6:** Effect of silymarin liposomes on SOD, GSH-PX, and MDA in experimental hepatitis animal models induced by CCl_4_ ($\bar{x}$±s, n = 10).

Group	Dose	SOD	GSH-PX	MDA
	(mg/kg.d)	(U/mgprot)	(U/mgprot)	(nmol/mgprot)
Blank group	—	81.02±10.14	184.78±33.24	1.29±0.34
Model group	—	53.56±10.04^◆◆^	78.74±23.20^◆◆^	1.99±0.61^◆◆^
Silymarin tablets	75	52.05±14.14	89.21±32.26	1.62±0.44
Silymarin tablets	150	55.07±12.05	94.21±20.95	1.63±0.21
Silymarin tablets	300	57.07±9.82	107.93±16.38^★^	1.61±0.21
Silymarin liposomes	75	63.00±8.80	115.56±21.20^★^	1.69±0.36
Silymarin liposomes	150	66.82±12.21	147.40±20.13^★★^ ^▲▲^	1.58±0.23
Silymarin liposomes	300	72.46±8.98^★★^ ^▲^	167.12±23.02^★★^ ^▲▲^	1.27±0.20^★★^

*vs* the blank group

^◆◆^ ***P*<0.01; *vs* the model group**

^★^ ***P*<0.05,**

^★★^ ***P*<0.01;**

*vs* silymarin tablets

^▲^ ***P*<0.05,**

^▲▲^ ***P*<0.01.**

**Table 7:** Effect of silymarin liposomes on SOD, GSH-PX, and MDA in the experimental hepatitis animal model induced by D-GalN ($\bar{x}$±s, n = 10).

Group	Dose	SOD	GSH-PX	MDA
	(mg/kg.d)	(U/mgprot)	(U/mgprot)	(nmol/mgprot)
Blank group	—	101.42±19.99	193.76±31.42	2.38±0.58
Model group	—	58.87±13.12^◆◆^	96.57±26.96^◆◆^	6.49±1.07^◆◆^
Silymarin tablets	75	66.32±12.79	103.63±31.00	6.06±2.01
Silymarin tablets	150	77.31±22.24^★^	112.52±39.28	5.03±2.01
Silymarin tablets	300	79.48±15.10^★^	123.57±34.22	4.87±1.06
Silymarin liposomes	75	86.85±22.42^★★^ ^▲^	135.91±45.02	4.66±2.84
Silymarin liposomes	150	89.80±14.47^★★^	153.71±28.54^★★^ ^▲^	3.59±1.45^★★^
Silymarin liposomes	300	95.90±15.68^★★^	176.24±22.78^★★^ ^▲▲^	3.10±0.76^★★^

*vs* the blank group

◆◆***P*<0.01; *vs* the model group,**

★***P*<0.05,**

★★***P*<0.01;**

*vs* silymarin tablets

▲***P*<0.05;**

▲▲***P*<0.01.**

**Table 8:** Effect of silymarin liposomes on SOD, GSH-PX, MDA, and NO in the experimental hepatitis animal model induced by BCG+LPS ($\bar{x}$±s, n = 10).

Group	Dose	SOD	GSH-PX	MDA	NO
	(mg/kg.d)	(U/mgprot)	(U/mgprot)	(nmol/mgprot)	(nmol/mgprot)
Blank group	—	114.63±29.88	187.40±29.34	2.22±0.60	1.06±0.48
Model group	—	77.23±13.63^◆◆^	115.95±28.13^◆◆^	5.39±2.08^◆◆^	4.51±1.31^◆◆^
Silymarin tablets	75	84.77±12.38	131.80±26.22	5.28±2.02	3.99±1.00
Silymarin tablets	150	92.63±14.78	160.05±22.18^★★^	3.52±1.45^★★^	2.95±1.43^★★^
Silymarin tablets	300	103.27±10.77^★^	168.20±19.82^★★^	2.97±0.93^★★^	1.64±0.63^★★^
Silymarin liposomes	75	101.1	172.13	3.16	2.35
		±14.72^★^	±22.00^★★^ ^▲▲^	±0.69^★★^ ^▲▲^	±1.19^★★^ ^▲▲^
Silymarin liposomes	150	103.19±17.82^★^	170.75±27.51^★★^	2.60±0.50^★★^	2.07±0.66^★★^
Silymarin liposomes	300	100.66±20.29^★^	178.32±24.73^★★^	2.98±1.00^★★^	1.21±0.72^★★^

*vs* the blank group

^**◆◆**^ ***P*<0.01; *vs* the model group,**

^**★**^ ***P*<0.05,**

^**★★**^ ***P*<0.01;**

*vs* silymarin tablets

^**▲▲**^ ***P*<0.01.**

## Conclusion

We have successfully prepared a new silymarin proliposomal formulation that can be scaled up easily for large-scale production. The drug-to-phospholipid ratio and pH affected the encapsulation efficiency of silymarin into the liposomes. A significant increase in the entrapment efficiency was achieved when the drug-to-phospholipid mass ratio or pH was increased. In this study, even though the entrapment efficiency is somewhat low, we can still prove that this method can be used to prepare effective liposomes.

According to the simple everted rat sac transport experiments, silymarin liposome transport across the intestinal barrier is better than for a silymarin solution, which shows that silymarin liposomes are a potential delivery system for silymarin to improve its bioavailability in vivo. Positive hepatoprotective effects demonstrated that silymarin is an effective hepatoprotective drug and that silymarin liposomes can improve these hepatoprotective effects. An obvious protective effect for experimental liver damage was achieved. The results of this study show that this proliposome formulation can serve as an efficient oral drug-delivery system for silymarin.

## Supporting Information

S1 Table Raw data of entrapment efficiency of liposomes and everted rat intestinal sac transport results. (XLS)

S2 Table Raw data of the hepatoprotective effects of silymarin liposomes on experimental hepatitis induced by CCL4. (XLS)

S3 Table Raw data of the hepatoprotective effects of silymarin liposomes on experimental hepatitis induced by D-GalN. (XLS)

S4 Table Raw data of the hepatoprotective effects of silymarin liposomes on experimental hepatitis induced by BCG+LPS. (XLS)

## References

[1] Ghosh A, Ghosh T, Jain S. Silymarin.-A review of the pharmacodynamics and bioavailability enhancement approaches. J Pharm Sci and Tech 2012;2:348–55.

[2] Theodosiou E, Purchartová K, Stamatis H, Kolisis F, Křen V. Bioavailability of silymarin flavonolignans: drug formulations and biotransformation. Phytochem Rev 2014;13:1–18.

[3] Li X, Yuan Q, Huang Y, Zhou Y, Liu Y. Development of silymarin self-microemulsifying drug delivery system with enhanced oral bioavailability. AAPS Pharm Sci Tech 2010;11:672–78. 10.1208/s12249-010-9432-x PMC2902333 20405254

[4] Sonali D, Tejal S, Vaishali T, Tejal G. Silymarin-solid dispersions: characterization and influence of preparation methods on dissolution. Acta Pharm 2010;60:427–43. 10.2478/v10007-010-0038-3 21169135

[5] Barzaghi N, Crema F, Gatti G, Pifferi G, Perucca E. Pharmacokinetic studies on IdB1016, a silybin- phosphatidylcholine complex, in healthy human subjects. Eur J Drug Metab Pharmacokinet 1990;15:338–8. 10.1007/BF03190223 2088770

[6] Zhang Y, Li J. Preparation and in vitro release of silymarin-loaded polyethylene glycol modified liposomes, pharmaceutical and clinical. Resources 2010;18:239–42.

[7] Elmowafy M, Viitala T, Ibrahim HM, Abu-Elyazid SK, Samy A, Kassem A. et al Silymarin loaded liposomes for hepatic targeting: in vitro evaluation and HepG2 drug uptake. Eur J Pharm Sci 2013;50:161–71. 10.1016/j.ejps.2013.06.012 23851081

[8] Wu W, Wang Y, Que L. Enhanced bioavailability of silymarin by self-microemulsifying drug delivery system. Eur J Pharm Biopharm 2006;63:288–94. 16527467 10.1016/j.ejpb.2005.12.005

[9] Liu LJ, Pang XJ, Zhang W, Wang S. Formulation design and in vitro evaluation of silymarin-loaded self-microemulsifying drug delivery systems. Asian J Pharm 2007;2:150–60.

[10] Parveen R, Baboota S, Ali J, Ahuja A, Vasudev SS, Ahmad S. Oil based nanocarrier for improved oral delivery of silymarin: in vitro and in vivo studies. Int J Pharm 2011;413:245–53. 10.1016/j.ijpharm.2011.04.041 21549187

[11] Cao X, Fu M, Wang L, Liu H, Deng W, Qu R. et al Oral bioavailability of silymarin formulated as a novel 3-day delivery system based on porous silica nanoparticles. Acta Biomater 2012;8:2104–12. 10.1016/j.actbio.2012.02.011 22343518

[12] Maherani B, Arab-Tehrany E, R. Mozafari M, Gaiani C, Linder M. Liposomes: a review of manufacturing techniques and targeting strategies. Curr Nanosci 2011;7:436–52.

[13] Karn PR, Cho W, Hwang SJ. Liposomal drug products and recent advances in the synthesis of supercritical fluid-mediated liposomes. Nanomedicine (Lond) Nanomedicine (Lond) 2013;8:1529–48. 23987112 10.2217/nnm.13.131

[14] Kumar N, Rai A, Reddy ND, Raj PV, Jain P, Deshpande P. et al Silymarin liposomes improves oral bioavailability of silybin besides targeting hepatocytes, and immune cells. Pharmacol Rep 2014;66:788–98. 10.1016/j.pharep.2014.04.007 25149982

[15] Shaji J, Proliposomes BV. A brief overview of novel delivery system. Int J Pharm Bio Sci 2013;4:150–60.

[16] Wang J, Li MX. The development of the proliposome. J Chin Med Industry 2005;36:707–11.

[17] Potluri P, Betageri GV. Mixed-micellar Proliposomal systems for enhanced oral Delivery of progesterone. Drug Deliv 2006;13:227–32. 16556576 10.1080/10717540500395007

[18] Wagner A, Vorauer-Uhl K. Liposome Technology for Industrial purposes [Online] 2011. Available at: http://www.hindawi.com/journals/jdd/2011/591325/. Accessed on 20 October 2010. 10.1155/2011/591325 PMC3065896 21490754

[19] Sun C, Wang J, Liu J, Qiu L, Zhang W, Zhang L. Liquid Proliposomes of Nimodipine Drug Delivery system: preparation, characterization, and pharmacokinetics. AAPS PharmsciTech 2013;14:332–8. 10.1208/s12249-013-9924-6 23319300 PMC3581684

[20] Hildebrand A, Beyer K, Neubert R, Garidel P, Blume A. Temperature dependence of the interaction of cholate and deoxycholate with fluid model membranes and their solubilization into mixed micelles. Colloids Surf B Biointerfaces 2003;32:335–51.

[21] Kossena GA, Boyd BJ, Porter CJ, Charman WN. Separation and characterization of the colloidal phases produced on digestion of common formulation lipids and assessment of their impact on the apparent solubility of selected poorly water-soluble drugs. J Pharm Sci 2003;92:634–48. 12587125 10.1002/jps.10329

[22] Porter CJ, Trevaskis NL, Charman WN. Lipids and lipid-based formulations: optimizing the oral delivery of lipophilic drugs. Nat Rev Drug Discov 2007;6:231–48. 17330072 10.1038/nrd2197

[23] Gao XL, Kzybek M, Wen H. [In vivo distribution of a novel proliposomal preparation of tegafur following intragastric gavage to rats]. Yao Xue Xue Bao 2005;40:1139–43. 16496681

[24] Wang M, Gao XL. Determination of the entrapment efficiency of progesterone proliposome. Chin J New Drugs 2011;20:75–7.

[25] Chu C, Tong SS, Xu Y, Wang L, Fu M, Ge YR. et al Proliposomes for oral delivery of dehydrosilymarin: preparation and evaluation in vitro and in vivo. Acta Pharmacol Sin 2011;32:973–80. 10.1038/aps.2011.25 21666703 PMC4003120

[26] Niu M, Lu Y, Hovgaard L, Wu W. Liposomes containing glycocholate as potential oral insulin delivery systems:preparation, in vitro evaluation and improved protective effect of cholate type, particle size and administered dose. Eur J Pharm, Biopharm 2011;81:265–72.

[27] Hiremath PS, Soppimath KS, Betageri GV. Proliposomes of exemestane for improved oral delivery: formulation and in vitro evaluation using PAMPA, Caco-2 and rat intestine. Int J Pharm 2009;380:96–104. 10.1016/j.ijpharm.2009.07.008 19616608

[28] Alam MA, Al-Jenoobi FI, Al-mohizea AM. Everted gut sac model as a tool in pharmaceutical research: limitations and applications. J Pharm Pharmacol 2012;64:326–36. 10.1111/j.2042-7158.2011.01391.x 22309264

[29] El-Samaligy MS, Afifi NN, Mahmoud EA. Evaluation of hybrid liposomes-encapsulated silymarin regarding physical stability and in vivo performance. Int J Pharm 2006;319:121–29. 16837151 10.1016/j.ijpharm.2006.04.023

[30] Chen Y, Lu Y, Chen J, Lai J, Sun J, Hu F. et al Enhanced bioavailability of the poorly water-soluble drug fenofibrate by using liposomes containing a bile salt. Int J Pharm 2009;376:153–60. 10.1016/j.ijpharm.2009.04.022 19394416

[31] Rao S, Tan A, Thomas N, Prestidge CA. Perspective and potential of oral lipid-based delivery to optimize pharmacological therapies against cardiovascular diseases. J Control Release 2014;193:174–87. 10.1016/j.jconrel.2014.05.013 24852093

